# Quality of life of patients with rheumatic diseases during the COVID-19 pandemic: The biopsychosocial path

**DOI:** 10.1371/journal.pone.0262756

**Published:** 2022-01-18

**Authors:** Guillermo A. Guaracha-Basáñez, Irazú Contreras-Yáñez, Gabriela Hernández-Molina, Viviana A. Estrada-González, Lexli D. Pacheco-Santiago, Salvador S. Valverde-Hernández, José Roberto Galindo-Donaire, Ingris Peláez-Ballestas, Virginia Pascual-Ramos

**Affiliations:** 1 Department of Immunology and Rheumatology, Instituto Nacional de Ciencias Médicas y Nutrición Salvador-Zubirán (INCMyN-SZ), Mexico City, Mexico; 2 Rheumatology Unit, Hospital General de México “Dr. Eduardo Liceaga”, Mexico City, Mexico; Jawaharlal Institute of Postgraduate Medical Education and Research, INDIA

## Abstract

**Background:**

Previous models that assess quality-of-Life (QoL) in patients with rheumatic diseases have a strong biomedical focus. We evaluated the impact of COVID-19 related-health care interruption (HCI) on the physical, psychological, social relationships and environment QoL-dimensions, and explored factors associated with QoL when patients were reincorporated to the outpatient clinic, and after six-month follow-up.

**Patients and methods:**

Study phase-1 consisted of a COVID-19 survey administered from June 24^th^-October 31^st^ 2020, to outpatients with rheumatic diseases who had face-to-face consultation at outpatient clinic reopening. Study phase-2 consisted of 3 consecutive assessments of patient´s QoL (WHOQOL-BREF), disease activity/severity (RAPID-3), and psychological comorbidity/trauma (DASS-21 and IES-R) to patients from phase-1 randomly selected. Sociodemographic, disease and treatment-related information, and comorbidities were obtained. Multiple linear regression analysis identified factors associated with the score assigned to each WHOQOL-BREF dimension.

**Results:**

Patients included (670 for phase-1 and 276 for phase-2), had primarily SLE and RA (44.2% and 34.1%, respectively), and all the dimensions of their WHOQOL-BREF were affected. There were 145 patients (52.5%) who referred HCI, and they had significantly lower dimensions scores (but the environment dimension score). Psycho-emotional factors (primarily feeling confused, depression and anxiety), sociodemographic factors (age, COVID-19 negative economic impact, years of scholarship, HCI and having a job), and biomedical factors (RAPID-3 score and corticosteroid use) were associated with baseline QoL dimensions scores. Psycho-emotional factors showed the strongest magnitude on dimensions scores. Most consistent predictor of six-month follow-up QoL dimensions scores was each corresponding baseline dimension score, while social determinants (years of scholarship and having a job), emotional factors (feeling bored), and biomedical aspects (RAPID 3) had an additional impact.

**Conclusions:**

HCI impacted the majority of patient´s QoL dimensions. Psycho-emotional, sociodemographic and biomedical factors were consistently associated with QoL dimensions scores, and these consistently predicted the QoL trajectory.

## Background

Quality-of-life (QoL) is a multidimensional construct, open to various definitions, approaches, and ideological uses [1–4]. The World Health Organization Quality of Life (WHOQOL) Group defines the QoL as a construct that encompasses individuals’ perceptions of their position in life, in the context of the culture and value systems in which they live, and concerning their goals, expectations, standards, and concerns [5]. This definition reflects that QoL refers to a subjective evaluation and that its use varies from individual to individual. However, most people will positively connotate the term, which possesses this sense of personal goodness and conjures up pleasant notions of how we want to be and how we want to live [1–3].

Patients with rheumatic diseases have a significantly impaired (health-related) QoL (HRQoL); disease activity, comorbidities, and treatment-related side effects are some of the contributors to the decrease functioning that extends to physical, emotional, and social dimensions [6–9]. In the field of rheumatic diseases, HRQoL measures drive clinical decisions and add value to cost-utility analyses [10, 11]. Despite this, few rheumatologists use QoL measures in their clinical practices, even though surveys indicate that most perceive these measures as valuable [7, 12]. Previous models of outcomes for rheumatic conditions have a strong biomedical focus, having the implicit assumption that there is a linear relationship between disease processes and patient’s QoL [8]. However, newer approaches to examining patient-reported outcomes acknowledge the roles of demographic, physiological, psychological, social, and environmental factors acting as buffers and triggers of poor outcomes [13, 14]. In such context, generic measures of QoL identify associations between physical conditions and mental health and highlight the need to address psychological functioning to ultimately acquire a comprehensive knowledge of individuals’ QoL [8].

The Coronavirus Disease 2019 (COVID-19) pandemic has emerged as an unprecedented challenge to health care systems and patients with chronic conditions [15]. Partial or complete closure of outpatient clinics has been implemented in many countries, which has negatively impacted the management and disease course of rheumatic diseases [16–19]. In addition, negative emotions, psychological conditions, and changes in patient’s behavior, such as non-adherence to medication, had already been described in a substantial number of rheumatic patients [20–28] and recognized as risk factors for the poor QoL [20, 27]. However, in previous studies, QoL assessment has been reduced to physical and psychological health, limiting the comprehensiveness of the topic.

We previously showed that health care interruption (HCI) during the COVID-19 pandemic impacted the clinical status of the underlying rheumatic disease, which was assessed from the physician’s perspective, among 670 patients with different rheumatologic diagnoses [29]. To approach the patient’s perspective, we evaluated the impact of HCI on four dimensions of patients’ QoL- the physical, psychological, social relationships and environment dimensions, and two additional facets- overall QoL and general health; we also explored factors associated to the QoL when patients were reinstalled at the outpatient clinic, and after six months of continuous follow-up.

## Patients and methods

### Ethics

The study was registered in clinicaltrials.gov (NCT04557358) and performed in compliance with the Helsinki Declaration [30]. The Research Ethics Committee of the Instituto Nacional de Ciencias Médicas y Nutrición Salvador Zubirán approved the study (reference number: IRE-3467). Written informed consent was obtained from all the patients.

### Study characteristics and target population

**Fig 1:**
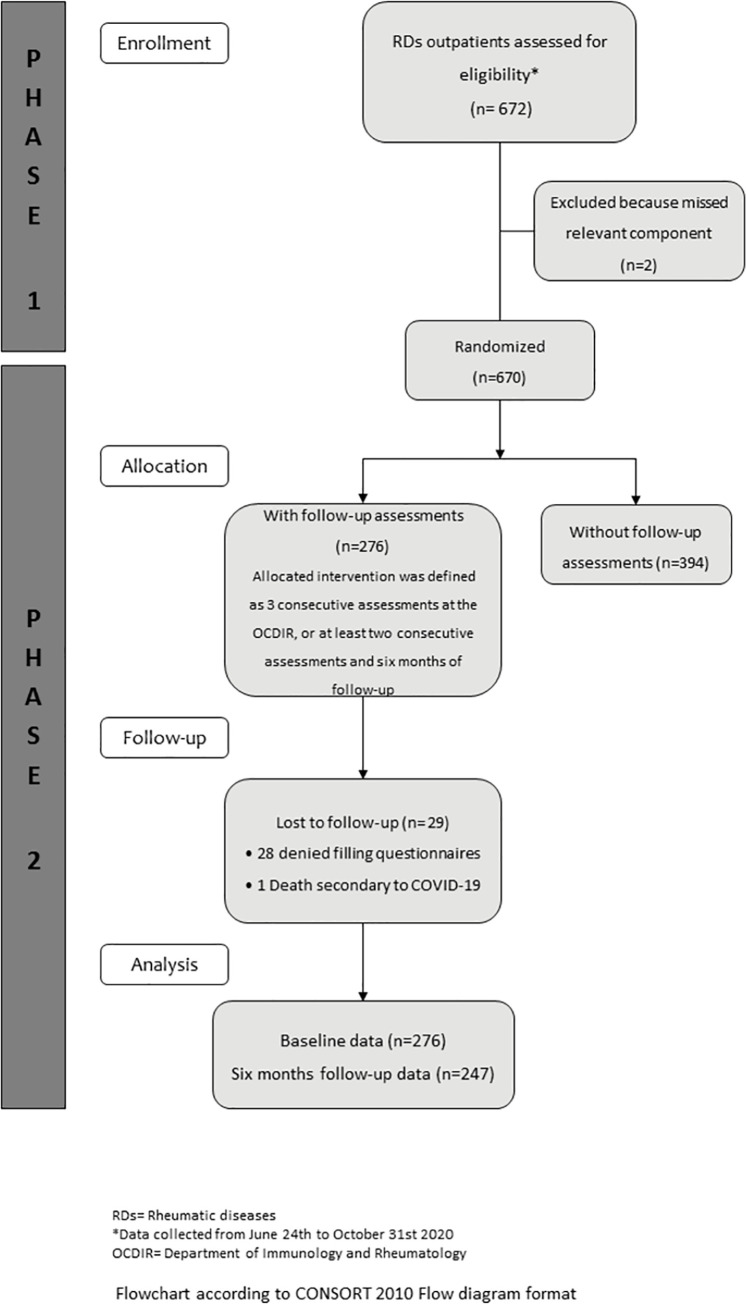
Study flowchart. Fig 1 summarizes phase 1 and phase 2 flowchart according to CONSORT 2010 flow diagram format.

The study was prospective and developed in two phases at the outpatient clinic of the Department of Immunology and Rheumatology (OCDIR) of a national referral center for rheumatic diseases (Fig 1 and S1 Checklist). In March 2020, the Institution was declared a dedicated COVID-19 hospital, in-person visits to the OCDIR were interrupted and, when possible, moved to phone consultations. In June 2020, the OCDIR was partially reinstalled.

*Phase 1* consisted of a survey administered from June 24^th^ to October 31^st^ to 670 outpatients with a definite rheumatic disease who had a face-to-face consultation when the OCDIR was reinstalled. These patients corresponded to 90% of the patients with a scheduled consultation. Survey development involved a multidisciplinary group that agreed on five components to be included, on individual items and their scale responses, and their distribution into the five components; survey validation was performed by eleven experts who determined face and content validity, and 40 outpatients who participated in pilot testing. The survey five components were (Please refer to the S1 Appendix): patient’s HCI (Yes/No) and reasons (two items), patient’s need for medical care and hospitalization during HCI (three items), patient’s need for communication with attending physicians or additional healthcare professionals (six items), patients modification of rheumatic disease-related treatment and reasons (three items) and patients perception of risk for SARS-CoV-2 infection (16 items, adapted from [31]). Relevant survey results had been previously described [29].

*Phase 2* (*follow-up*) consisted of 3 consecutive standardized face-to-face clinical assessments at the OCDIR (or at least two consecutive assessments and six months of follow-up) to 276 patients, randomly selected from the 670 patients in whom the survey was administered during phase 1. The baseline evaluation of phase 2 coincided with survey application (in the 276 patients randomized), and their follow-up clinical assessments were scheduled three to six months apart, depending on the patient’s disease activity status. Also, the six-month follow-up was deemed a convenient lag time to identify improvement in relevant outcomes.

During the study period, consecutive outpatients with a definite rheumatologic diagnosis according to the attendant rheumatologist criteria were invited to participate. Exclusion criteria included patients lost to follow-up during the pre-pandemic era, patients referred for the first time to the OCDIR, and patients with uncontrolled and severe comorbid conditions that might preclude treatment of the underlying rheumatic disease.

### Patient’s assessments

All the patients who agreed to participate had the following data retrieved on standardized formats: Sociodemographic information (age, sex, years of scholarship, household type, labor information, access to Social Security benefits and socioeconomic level), disease and treatment-related information (specific rheumatic diagnosis, disease duration, and corticosteroid, immunosuppressive drugs and antimalarial use) and comorbid conditions according to the Rheumatic Disease Comorbidity Index [32]. In addition, the following patient-reported outcomes were obtained at baseline and follow-ups (phase 2), and Spanish validated versions of the instruments were used: Quality of Life as per the WHOQOL-BREF [5], physical function, pain, and a patient global estimate evaluation of the underlying rheumatic disease as per the Routine Assessment of Patient Index Score-3 (RAPID-3) [33], and psychological comorbidity/trauma as per the Depression Anxiety Stress Scale-21 (DASS-21) [34] and the Impact of Event Scale-Revised (IES-R), [35, 36].

Also, attendant rheumatologists (21 assigned to the OCDIR) scored the patient’s clinical status according to the current level of disease activity, the course of disease activity, and the rheumatic disease control [29].

Finally, at baseline (and six-month follow-up) patient’s risk perception for the SARS-CoV-2 infection component survey was applied [29, 31]. The component was part of the COVID-19 survey (S1 Appendix).

### Measurements

#### WHOQOL-BREF

The WHOQOL-BREF included 26 items and was derived from data collected using the WHOQOL-100 items. It produces scores for four domains related to the QoL: physical health, psychological health, social relationships, and environment. It also includes one facet of overall QoL and general health (two additional items). Each domain score can be transformed to a 0–100 scale, with higher scores translating into a better QoL. The overall QoL and general health facets are scored on a five-point Likert scale and are presented from one to five, with higher scores translating into better outcomes.

#### RAPID-3

The RAPID-3 includes three measures: Physical function, pain, and a patient global estimate evaluation. It has a raw score of 0–30 and an adjusted score of 0–10, with higher scores translating into higher disease activity/severity. Four proposed categories are defined based on 0–30 scale cut-offs: >12 high, 6.1–12.0 as moderate, 6.0–3.1 as low, and ≤three near-remission.

#### DASS-21

The scale is a set of three self-reported subscales designed as a screening tool to assess the core symptoms of depression, anxiety, and stress. The three DASS-21 subscales contain seven items that are rated on a four-point Likert scale ranging from zero ("Did not apply to me at all") to three ("Applied to me very much, or most of the time"). The depression subscale assesses dysphoria, hopelessness, devaluation of life, self-deprecation, lack of interest/involvement, anhedonia, and inertia. The anxiety subscale assesses autonomic arousal, skeletal muscle effects, situational anxiety, and subjective experience of anxious affect. The stress subscale assesses difficulty relaxing, nervous arousal, and being easily upset/agitated, irritable/over-reactive, and impatient. Scores for depression, anxiety, and stress are calculated by summing the scores for the relevant items of each subscale. The DASS-21 is based on a dimensional rather than a categorical conception of psychological disorder and may have no direct implication with diagnostic categories in traditional classificatory systems; however, recommended cut-off scores for conventional severity labels (normal, mild, moderate, severe, and highly severe) have been published [37].

#### IES-R

The scale is a 22-item self-reported measure that assesses subjective distress caused by traumatic events. It is a revised version of the older version, the 15-item IES. The IES-R contains seven additional items related to the hyperarousal symptoms of post-traumatic stress disorder, which were not included in the original IES. Respondents are asked to identify a specific stressful life event and then indicate how much they were distressed or bothered during the past seven days by each "difficulty" listed. For this research, the stressful life event was standardized as the current pandemic. Items are rated on a five-point Likert scale ranging from zero ("Not at all") to four ("Extremely"). The IES-R yields a total score, from 0 to 88, with higher scores translating into more severe distress. A score ≥ of 33 indicates Post-Traumatic Stress Disorder [35, 36], which was conceptualized in the current manuscript plainly as "Post-traumatic Stress" due to the lack of diagnostic validation with a psychiatric interview.

#### Patient’s perception of risk for SARS-CoV-2 infection component (S1 Appendix)

The component included 16 items distributed as follows: Patient’ perception of the pandemic seriousness in the country (one item, five-point Likert scale), patient’ risk perception of SARS-CoV-2 infection (one item, six-point Likert scale), patient’ follow-up of physical distancing recommendation (one item, five-point Likert scale), patient’ risk perception of SARS-CoV-2 infection at their reincorporation to the OCDIR (one item, six- point Likert scale), family economic impact attributed to COVID-19 pandemic (one item, positive/negative economic impact options), COVID-19 impact on the family-members relationship (one item, Negative impact/Without impact/Positive impact), and ten patients’ negative emotions attributed to the COVID-19 pandemic (feeling- anxious, worried, fearful, alertness, depressed, confused, alarmed, isolated, discriminated against and bored; each one was rated on a five-point Likert scale, according to symptom’s intensity from “None” to “Very much”).

### Sample size calculation and selection of the follow-up sample

To detect an effect size (d) of 0.876 for the absolute difference in at least one of the dimension scores of the WHOQOL-BREF between patients with and without HCI, we estimated the sample size using a two-tailed test, a 5% significance level, and a power of 95%. The magnitude of the positive [19] difference was based on the smallest minimal clinically significant difference in the different dimensions of the WHOQOL-BREF, reported by De Mol et al. [38]. The G*Power estimate was a total sample size of 84 patients: 40 included in patients with HCI and 44 in the group without HCI, already accounting for 20% of losses.

Patients included in phase 2 study were randomly selected among the 670 patients included in phase 1 study, using randomly permuted blocks (software available at website www.randomization.com). Two groups of patients were defined, those included in phase 2 study (follow-up) and those not included.

Finally, we determined the power of the different models to explore factors associated with the QoL when patients were reinstalled at the outpatient clinic and after six months of continuous follow-up; in the different models tested, power was ≥ 95%.

### Statistical analysis

Descriptive statistics were used with frequencies and percentages for dichotomous variables and mean ± standard deviation (SD) or median (IQR) for continuous variables with normal and non-normal distribution, respectively.

HCI was defined as the cancelation of a scheduled face-to-face appointment to the OCDIR without re-scheduling within the next 3 months AND/OR care not provided to patients who required rheumatologic emergency care AND/OR patients’ decision not to attend the OCDIR; the outcome was based on the answer provided to the first component of the survey [29]. The baseline characteristics of patients with and without HCI were compared using the X^2^ test for the categorical variables, Student’s t-test for continuous variables with a normal distribution, and the Mann-Whitney U test for continuous variables with non-normal distribution.

The Rheumatic Disease Comorbidity Index scoring was modified, and when present, “depression” was omitted from the final score (four patients).

Stepwise forward multiple linear regression analysis was performed to identify factors associated with the score assigned to each dimension of the WHOQOL-BREF and the overall QoL and general health facet scores, which were considered dependent variables. Previously, we conceived a global model, driven by clinical experience from a multidisciplinary team, background knowledge derived from previous clinical research in populations with similar characteristics, and a biopsychosocial approach to patients with rheumatic diseases. This global model guided the initial selection of variables from the three spheres (biological, psycho-emotional, and social). The following factors were considered as simultaneous independent variables after collinearity was revised: Age (continuous), sex (Female/Male), years of scholarship (continuous), living together (Yes/No), formal and non-formal job (Yes/No), non-RA diagnosis (Yes/No), rheumatic disease duration (continuous), corticosteroid use (Yes/No), immunosuppressive drugs (Yes/No), comorbidities score (continuous), RAPID-3 score (continuous), each one of depression, anxiety, stress and post-traumatic stress (Yes/No; published cut-offs were used [37] with each specific construct considered to be present if severity label was at least moderate), four negative emotions that were not included in the former constructs- feeling bored, isolated, confused and discriminated against (Yes/No present; presence was considered if intensity was scored as Very much/Much), HCI (Yes/No), negative family economic impact attributed to COVID-19 pandemic (Yes/No), patient’ risk perception of SARS-CoV-2 infection (Yes/No; presence was defined if scored as Very high/High) and COVID-19 impact on the family-members relationship (continuous).

Missing data at random for negative emotions varied from 4.3% for feeling worried, to 11.6% for feeling discriminated. We imputed missing values by multiple imputations, using the linear regression procedure in SPSS (five imputed datasets were created). Variables included in the imputation models were the negative emotions (left), patients’ age and years of scholarship, and WHOQOL-BREF dimensions and facets scores.

Linear regression models for six months follow-up dependent variables additionally included each correspondent baseline QoL dimension/facet score.

All statistical analyses were performed using SPSS (version 21.0, IBM Corp., Armonk, NY, USA) and STATA (version 14.0, Stata Corp LLC, College Station, TX). A value of p <0.05 was considered statistically significant.

## Results

### Population characteristics

There were 276 patients randomly selected who completed study phase 1 and were invited to enter study phase 2. All of them completed their baseline evaluation, while 247 patients (89.5%) completed the six-month follow-up evaluation.

The most frequent diagnoses were as follows: Systemic Lupus Erythematous (SLE) in 122 patients (44.2%), Rheumatoid Arthritis (RA) in 94 patients (34.1%), Systemic Sclerosis in 11 patients (4%), and Systemic Vasculitis (SV) in 10 patients (3.6%).

Comparison of baseline characteristics among those who completed/did not complete the study identified the following statistically significant differences, as summarized in the S1 Table. Patients from the former group had lesser stress as per DASS-21, referred to lesser frequently feeling discriminated against and had lesser frequently negative family economic impact attributed to the COVID-19 pandemic.

**Fig 2:**
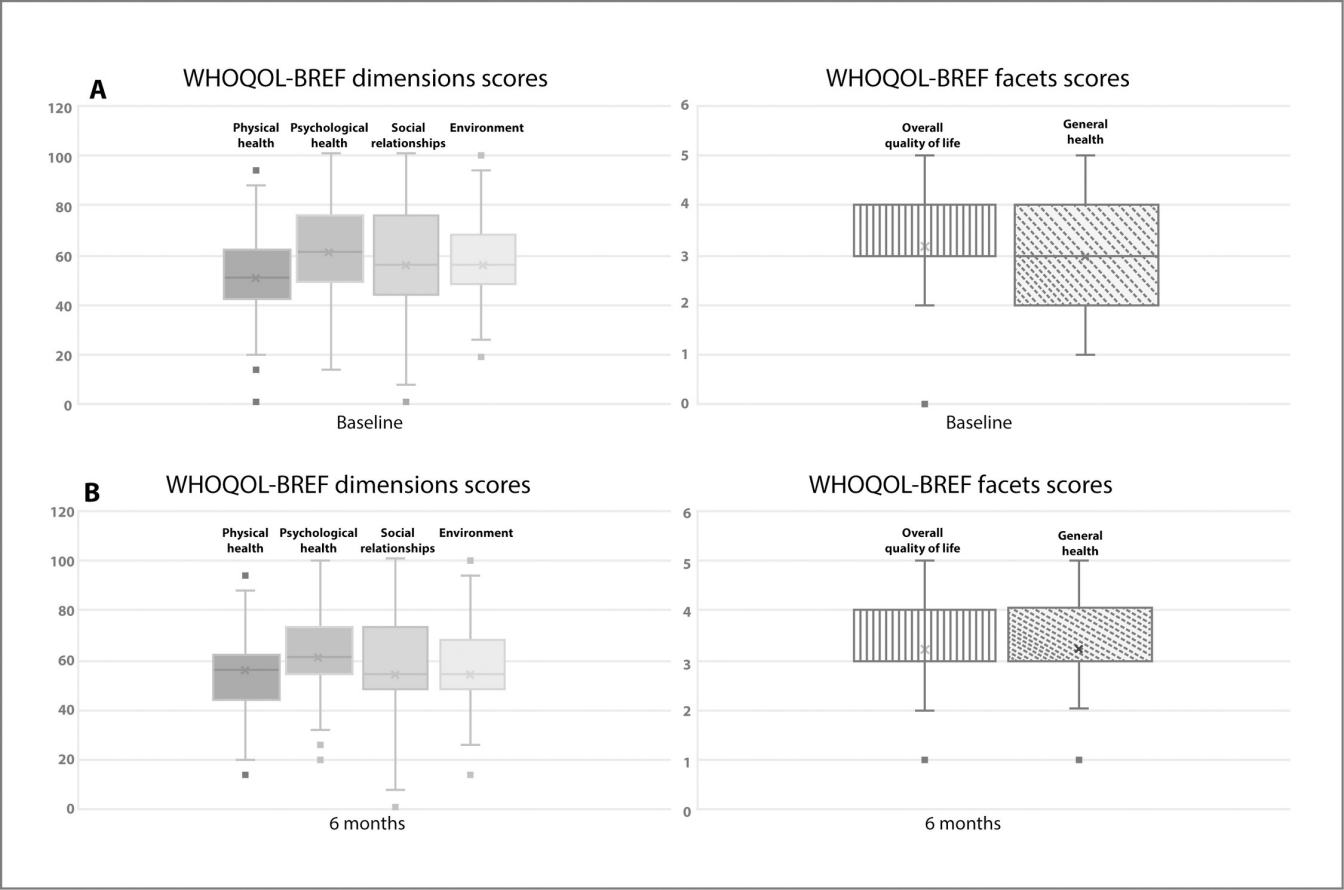
Baseline (Fig 2A) and six-month follow-up (Fig 2B) QoL dimensions/facets scores.

Tables 1 and 2 summarize population characteristics. Briefly, patients were primarily middle-aged females (229 [83%]), with (median, IQR) 12 (9–17) years of scholarship and middle-low socioeconomic level (249 [90.2%]). Almost half of the patients were living together (131 [47.5%]) and working (125 [45.3%]). Patients had substantial disease duration of the underlying rheumatic disease, and 202 (73.2%) were on immunosuppressive drugs, while 138 (50%) received a corticosteroid. Patients had adequate control of the rheumatic disease according to physician evaluation (178 [64.5%]), which was in accordance with the RAPID-3 score. Also, 106 (38.4%) patients had comorbid conditions, while 34–51 patients (12.3%-18.5%) had psychological comorbidity/trauma; meanwhile, negative emotions ranged from 25 (9.1%) patients feeling discriminated against to 168 (60.9%) patients feeling alert. The majority of the patients had (Very high/High) perception of the pandemic seriousness in the country (264 [96.4%]), followed (Always/Most of the time) physical distance recommendation (249 [90.2%]), and referred a negative economic impact of the pandemic (214 [78.1%]); almost half of the patients referred (Very high/High) risk perception of SARS-CoV-2 infection (133 [48.5%]). Finally, all the dimensions and facets of the patients’ QoL were compromised, particularly the physical health dimension, while the psychological health dimension showed the highest score (Fig 2).

**Table 1:** Characteristics of the population and their comparison in the subpopulations defined according to HCI/non-HCI: Socio-demography, rheumatic disease-related characteristics and QoL.

	Overall population	HCI	Non-HCI	p
	N = 276	N = 145 (52.5%)	N = 131 (47.5%)	
**Socio-demographic characteristics**				
Age, years	44 (33–55)	41 (30–52)	46 (36–57)	0.019
Females*	229 (83)	122 (84.1)	107 (81.7)	0.587
Years of scholarship	12 (9–17)	12 (9–17)	12 (9–17)	0.233
Living together*	131 (47.5)	62 (42.8)	69 (52.7)	0.100
Formal and non-formal job*	125 (45.3)	57 (39.3)	68 (51.9)	0.036
Access to Social Security benefits*	96 (34.8)	49 (33.8)	47 (35.9)	0.717
Middle-low socioeconomic level*	249 (90.2)	130 (89.7)	119 (90.8)	0.741
**Rheumatic disease characteristics**				
Non-RA diagnosis*	182 (65.9)	111 (76.6)	71 (54.2)	≤0.001
Disease duration, years	11 (6–19)	11 (4–19)	11 (7–19)	0.108
Corticosteroid use*	138 (50)	85 (58.6)	53 (40.5)	0.003
Immunosuppressive drug use*	202 (73.2)	110 (75.9)	92 (70.2)	0.291
Antimalarial use*	102 (37)	53 (36.6)	49 (37.4)	0.883
Rheumatic disease comorbidity index score	0 (0–1)	0 (0–1)	0 (0–1)	0.013
Rheumatic disease comorbidity index score ≥1*	106 (38.4)	66 (45.5)	40 (30.5)	0.011
Substantial disease activity level*^1^	48 (17.4)	31 (21.4)	17 (13)	0.066
Clinical deterioration*	64 (23.2)	34 (23.4)	30 (22.9)	0.914
Adequate control of the rheumatic disease*	178 (64.5)	89 (61.4)	89 (67.9)	0.255
RAPID-3 score	6.1 (1.5–11.8)	7.7 (2.3–13.2)	2.9 (1–9.5)	≤0.001
**Baseline QoL dimension/facets scores**				
Physical health dimension score (0–100)	53 (44–63)	50 (38–63)	56 (44–69)	0.001
Psychological health dimension score (0–100)	63 (50–75)	56 (50–69)	69 (56–75)	0.005
Social relationships dimension score (0–100)	56 (46–75)	56 (44–75)	56 (50–75)	0.042
Environment dimension score (0–100)	56 (50–69)	56 (47–66)	56 (50–69)	0.357
Overall quality of life facet score (1–5)	3 (3–4)	3 (2–4)	3 (3–4)	0.012
General health facet score (1–5)	3 (2–4)	3 (2–3)	3 (3–4)	≤0.001
**Six-month follow-up QoL dimension/facets scores^2^**				
Physical health dimension score (0–100)	56 (44–63)	56 (44–63)	56 (44–69)	0.235
Psychological health dimension score (0–100)	63 (56–75)	63 (50–70)	69 (56–75)	0.163
Social relationships dimension score (0–100)	56 (50–75)	56 (50–75)	56 (50–75)	0.370
Environment dimension score (0–100)	56 (50–69)	56 (50–69)	56 (50–69)	0.573
Overall quality of life facet score (1–5)	3 (3–4)	3 (3–4)	3 (3–4)	0.375
General health facet score (1–5)	3 (3–4)	3 (3–4)	3 (3–4)	0.060

*Number (%) of patients, data presented as median (IQR) unless otherwise indicated. RA = Rheumatoid Arthritis. HCI = Health Care Interruption. RAPID-3 score = Routine Assessment of Patient Index Score-3. QoL = Quality of Life.

^1^Patients with at least moderate disease activity level according to physician evaluation.

^2^There were 247 patients with 6 months follow-up information, among whom 130 had HCI (52.6%).

**Table 2:** Characteristics of the population and their comparison in the subpopulations defined according to HCI/non-HCI: Psychological comorbidity, negative emotions and patient´ perception of risk for SARS-CoV-2 infection component survey.

	Overall population	HCI	Non-HCI	p
	N = 276	N = 145 (52.5%)	N = 131 (47.5%)	
**Psychological comorbidity**				
Depression subscale score	1 (0–4)	2 (0–4)	1 (0–3)	0.002
Depression*	34 (12.3)	22 (15.2)	12 (9.2)	0.129
Anxiety subscale score	1 (0–4)	2 (0–6)	1 (0–3)	0.001
Anxiety*	51 (18.5)	38 (26.2)	13 (9.9)	0.001
Stress subscale score	3 (1–7)	5 (2–8)	2 (1–6)	≤0.001
Stress*	39 (14.1)	26 (17.9)	13 (9.9)	0.057
IES-R score	8 (3–21)	11 (4–26)	6 (2–16)	0.002
Post-traumatic distress*	36 (13.2)	24 (16.8)	12 (9.3)	0.069
**Negative emotions (Feeling Very much intensity/Much intensity)** *				
Anxious	102 (37)	66 (45.5)	36 (27.5)	0.003
Worried	138 (50)	84 (57.9)	54 (41.2)	0.008
Fearful	95 (34.4)	63 (43.4)	32 (24.4)	0.001
Alertness	168 (60.9)	94 (64.8)	74 (56.5)	0.175
Depressed	60 (21.7)	39 (26.9)	21 (16)	0.04
Confused	50 (18.1)	34 (23.4)	16 (12.2)	0.019
Alarmed	101 (36.6)	63 (43.4)	38 (29)	0.017
Isolated	134 (48.6)	74 (51)	60 (45.8)	0.401
Discriminated against	25 (9.1)	14 (9.7)	11 (8.4)	0.834
Bored	79 (28.6)	49 (33.8)	30 (22.9)	0.062
**Patient´ perception of risk for SARS-CoV-2 infection component survey** *				
(Very high/High) Patient’ perception of the pandemic seriousness in Mexico	264 (96.4)	140 (97.2)	124 (95.4)	0.418
(Very high/High) Patient’ risk perception of SARS-CoV-2 infection	133 (48.5)	68 (47.2)	65 (50)	0.646
(Always, most of the time) Patient´ follow-up of physical distance recommendation	249 (90.2)	134 (92.4)	115 (87.8)	0.196
(Very high/High) Patient’ risk perception of SARS-CoV-2 infection at their reincorporation to the OCDIR	116 (42.2)	61 (42.1)	55 (42.3)	0.968
Negative family economic impact attributed to COVID-19 pandemic	214 (78.1)	118 (82.5)	96 (73.3)	0.065
COVID-19 impact on the family-members relationship^1^	2 (2–3)	2 (1–3)	2 (2–3)	0.507

*Number (%) of patients, data presented as median (IQR) unless otherwise indicated. HCI = Health Care Interruption.

^1^From 1–3, where 1 = negative impact, 2 = neither positive nor negative impact, and 3 = positive impact.

### HCI and QoL

There were 145 patients (52.5%) who referred HCI, while 131 (47.5%) were not affected. Patients from the former group had significantly lower physical health and psychological health dimension scores, lower social relationships dimension scores, and lower overall QoL and general health facet scores than their counterparts. In contrast, environment dimension scores were similar between groups (Table 1).

Additional differences between both groups of patients are summarized in Tables 1 and 2. Patients with HCI were younger, referred lesser frequently a job, had more frequently a non-RA diagnosis, were more frequently on corticosteroid, and had higher RAPID-3 score than their counterparts. Also, they had more frequent comorbidities, scored higher DASS-21 subscales and the IES-R, and referred more frequently negative emotions (but feeling alert, isolated, discriminated against, and bored).

### Factors associated with baseline quality of life dimensions

**Fig 3:**
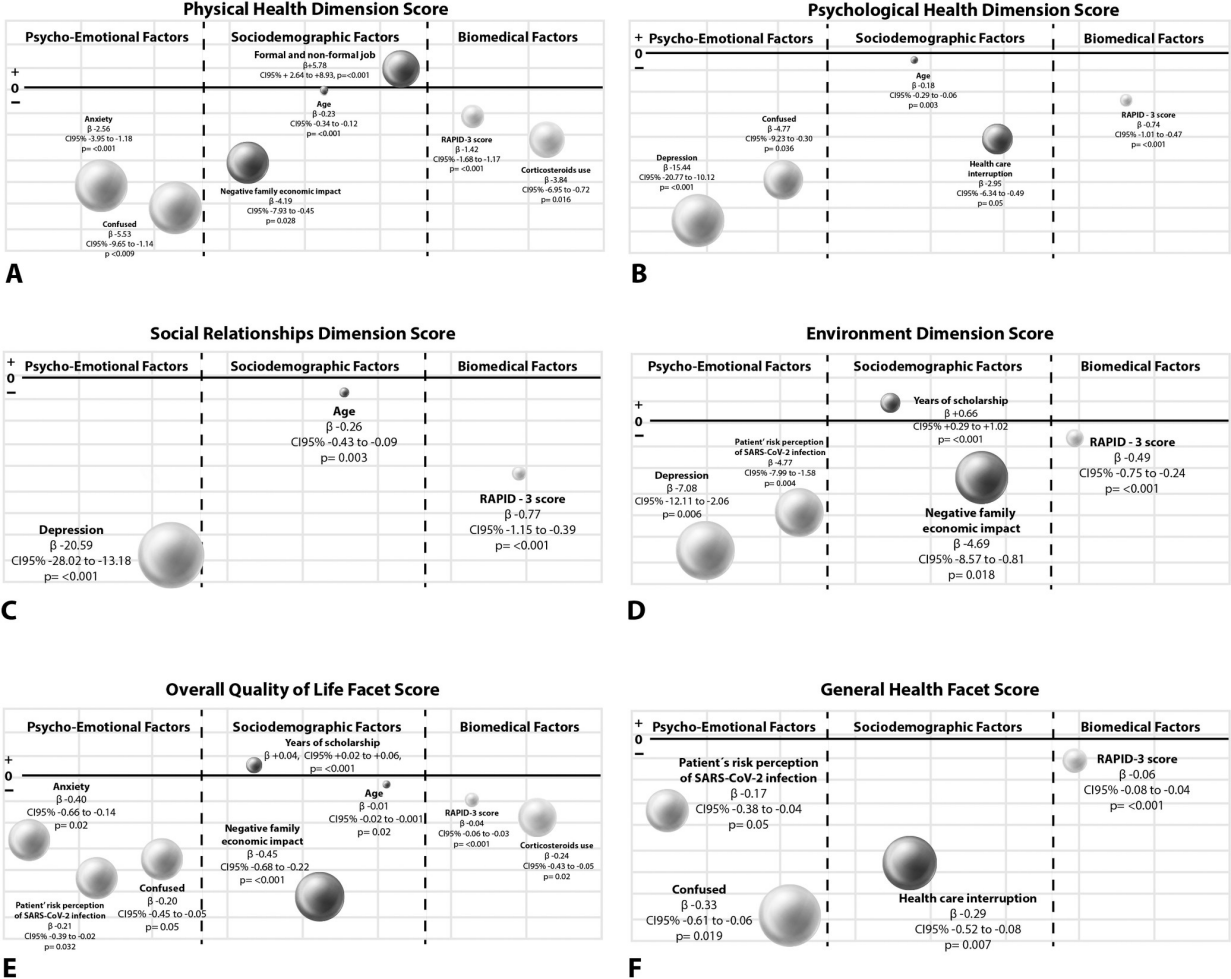
Factors associated with baseline QoL dimensions/facets scores: 3A Physical health dimension, 3B Psychological health dimension, 3C Social relationships dimension, 3D Environment dimension, 3E Overall QoL facet and 3F General health facet. Factors associated with QoL dimensions and facets scores are distributed into three columns. The column located at the left side of the figure includes psycho-emotional factors, while the column located at the right side includes biomedical factors. Sociodemographic factors are located in the column left. The size of each sphere is in accordance with B coefficient magnitude (multivariate linear regression analysis). Meanwhile, the space between spheres does not meant to represent any relevant data.

Fig 3A–3F summarizes results from multiple linear regression analysis. Feeling confused, anxiety, COVID-19 negative economic impact, corticosteroid use, RAPID-3 score, and age decreased the physical function dimension score while having a job increased the score (R^2^ = 0.53) (Fig 3A). Depression, feeling confused, HCI, RAPID-3 score, and age decreased the psychological function dimension score (R^2^ = 0.32), (Fig 3B), while depression, RAPID-3 score, and age decreased the social relationships dimension score (R^2^ = 0.21) (Fig 3C). Finally, depression, risk perception for SARS-Cov-2 infection, COVID-19 negative economic impact, and RAPID-3 score decreased the environment dimension score, while years of scholarship increased the score (R^2^ = 0.20) (Fig 3D).

Regarding WHOQOL-BREF facets, COVID-19 negative economic impact, risk perception for SARS-Cov-2 infection, feeling confused, anxiety, corticosteroid use, RAPID-3 score, and age decreased the QoL facet score. At the same time, years of scholarship increased the score (R^2^ = 0.34) (Fig 3E). Also, feeling confused, HCI, risk perception for SARS-Cov-2 infection and RAPID-3 score decreased the general health facet score (R^2^ = 0.24) (Fig 3F).

### Six-month QoL dimensions scores and predictors

Table 1 and Fig 2B summarized six months follow-up QoL dimensions/facets scores. Similar to baseline QoL dimension and facet scores, patients scored high on the psychological health dimension while having similar scores on the dimensions left, as did both facets. Comparison of six-month follow-up QoL outcomes between patients with/without HCI did not identify differences.

**Fig 4:**
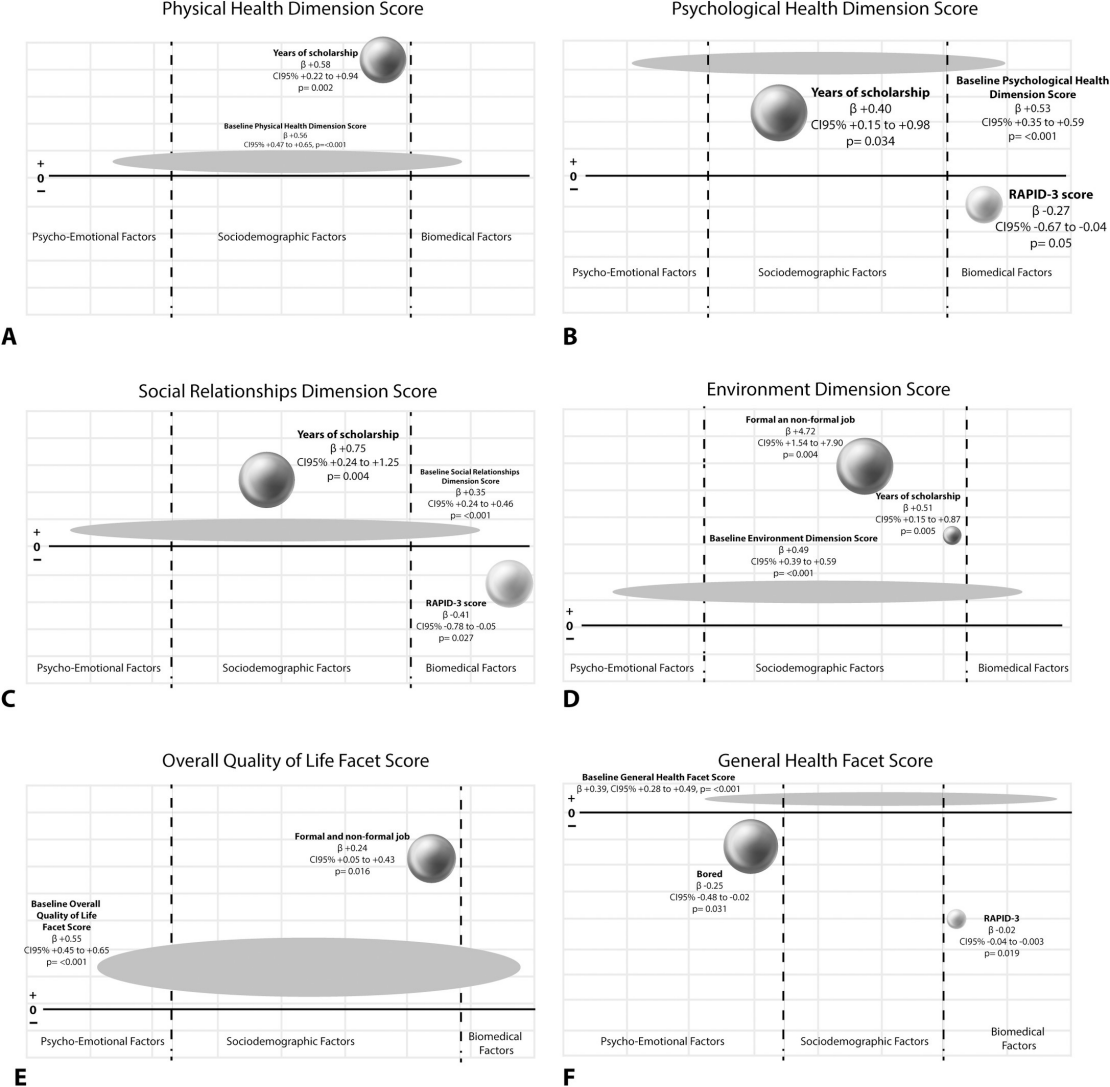
Factors associated with six-month follow-up QoL dimensions/facets scores: 4A Physical health dimension, 4B Psychological health dimension, 4C Social relationships dimension, 4D Environment dimension, 4E Overall quality of life facet and 4F General health facet. Factors associated with QoL dimensions and facets scores are distributed into three columns. The column located at the left side of the figure includes psycho-emotional factors, while the column located at the right side includes biomedical factors. Sociodemographic factors are located in the column left. The size of each sphere is in accordance with B coefficient magnitude (multivariate linear regression analysis). Meanwhile, the space between spheres does not meant to represent any relevant data.

Fig 4A–4F summarizes results from multiple linear regression analysis. Interestingly, the baseline dimension/facet score was a predictor for each corresponding six-month follow-up dimension/facet score. In addition, years of scholarship increased six-month follow-up physical health dimension score (R^2^ = 0.41) (Fig 4A); years of scholarship increased both, six-month follow-up psychological health dimension score and social relationship dimension score, while RAPID-3 score decreased each dimension score (R^2^ = 0.35, Fig 4B and R^2^ = 0.23, Fig 4C, respectively); years of scholarship and having a job increased six-month follow-up environment dimension score (R^2^ = 0.39, Fig 4D); having a job increased six-month follow-up overall QoL facet score (R^2^ = 0.35, Fig 4E); lastly, feeling bored and RAPID-3 score decreased the general health facet score (R^2^ = 0.28, Fig 4F).

## Discussion

Incorporating the term “quality-of-life” as a scientific concept into the medical literature is relatively recent. Engel’s development of a biopsychosocial model of medicine heralded the emergence of the scientific application of psychosocial concepts in medicine, and led to medical research embracing the QoL concept as a legitimate avenue of study [3, 39]. In rheumatic diseases, measurement of the QoL-related constructs has become increasingly important in clinical research and bedside clinic [9]. QoL measures have moved from secondary to primary endpoints in clinical trials and are currently considered predictors of relevant outcomes such as death [9, 40]. Published clinical practice guidelines recommend routinely evaluating patients’ QoL measures and using their assessment to modify and guide patient care [41], which has led to medical interventions currently designed to improve QoL rather than prolong the life [42, 43].

The current study showed that, during the COVID-19 pandemic, HCI affected one in two outpatients with rheumatic diseases in a tertiary care level center and COVID-19 dedicated hospital located in Mexico City. At patient reincorporation to the outpatient clinic, the physical-, emotional-, social relationship- and the environment- dimensions, and the QoL- and general health- facets of the patients’ QoL were compromised. However, patients affected by HCI scored lower on QoL dimensions and facets but the environment dimension. Overall, psycho-emotional factors (primarily feeling confused, depression and anxiety), sociodemographic factors (age, years of scholarship, having a job, COVID-19 negative economic family impact, and HCI), along with biomedical factors (RAPID-3 score) were consistently associated with baseline QoL dimensions and facets scores; however, their participation differed according to the dimension and facet evaluated. Finally, each corresponding baseline dimension/facet score was consistently associated with the six-month follow-up QoL dimensions and facets scores, while psycho-emotional, sociodemographic, and biomedical factors had a lesser relevant impact.

In the current study, we included the WHOQOL-BREF to comprehensively assess the QoL among patients with rheumatic diseases during the COVID-19 pandemic. The WHOQOL-BREF has good psychometric properties and is designed for a generic evaluation of four relevant dimensions of the QoL across many cultures; accordingly, it enables comparison with a wide range of diseases and conditions [44]. The WHOQOL-BREF encompasses a more significant number of domains that are integral to the assessment of the QoL. Notably, the social relationships and environment domains are not always included in other assessments; both domains/dimensions are crucial to assessing the overwhelming burden of the COVID-19 pandemic on individuals, including those with chronic conditions [45, 46].

First, the study showed that all the dimensions and facets of the patients’ QoL were compromised at patient’s reincorporation to the outpatient clinic, particularly the physical health dimension, followed by the social relationship and environment dimensions. In contrast, the psychological health dimension showed a better score. In addition, the negative impact of HCI on the disease activity course of the underlying rheumatic disease, which has been reported by different groups, including ours [19, 23, 29], extended to the different patient’s QoL dimensions and facets, but the environment dimension. The potential consequences of rheumatic diseases are so pervasive that every QoL dimension can be affected and might have an additive effect on those derived from the COVID-19 pandemic [45, 46]. Meanwhile, the environment dimension might have been deeply affected by the COVID-19 pandemic itself and the virus containment measures [45, 46], and the additional negative effect of HCI on the dimension might have been diluted.

Second, psycho-emotional, sociodemographic, and biomedical factors were consistently associated with baseline QoL dimensions and facets scores. Interestingly, psycho-emotional factors (primarily feeling confused, depression and anxiety) showed the most significant magnitude on dimensions and facets scores. Similar results were reported by Glintborg et al. [20] in more than 12 000 Danish patients with inflammatory rheumatic diseases, in whom high levels of anxiety and self-isolation persisted even after the Danish epidemic was well controlled; poor QoL, which was evaluated with the European QoL five dimensions (EQ-5D), was among the factors associated with both anxiety and self-isolation. Koppert TY et al. [28] examined the psychological impact of the peak of the COVID-19 crisis on 239 Dutch patients with an inflammatory rheumatic disease and 1821 controls. Patients from the former group were more worried and stressed during the peak of the COVID-19 pandemic, although their level of mental wellbeing was not reduced (compared to 2018); authors assessed mental wellbeing with the Dutch version of the RAND 36-item Short Form health survey and described a similar percentage to ours of their patients self-referred being worried/very worried with the peak period of COVID-19 pandemic. The association found between a negative emotion (feeling confused) and QoL dimension scores deserves some comments. Feeling confused might be considered a normal reaction in the context of the COVID-19 pandemic, where “infodemic” has characterized social media information. However, in some (very) confused patients, confusion might become excessive and impact mental health [47]. People use emotion-regulation strategies more regularly when they perceive an event as highly stressful, such as the case of a pandemic, where it may serve as a survival strategy [48]. Nonetheless, there are several mechanisms through which emotion-regulation occurs, and the selection of these strategies is a complex and non-uniform process that might impact mental disorders and wellbeing [49]; for example, the reappraisal of situations has been associated with positive emotion, general wellbeing and better interpersonal functioning, while suppressing them leads to more powerful negative emotions, worse social functioning and mental illness [50].

Third, the most relevant social factors associated with baseline dimensions and facets scores were age (consistently), COVID-19 negative economic impact, years of scholarship, HCI (limited to psychological function dimension and general health facet), and having a job. Guillemin et al [51] stated that disparities in care exist and affect people with chronic musculoskeletal conditions all around the world. Previous studies had also documented that people in society are exposed to inequity by demographic characteristics, societal factors, and living conditions, which determine to some degree the risk of disease, disability, and lower QoL; however, these factors are common to the general population, and therefore also apply to people with chronic rheumatic diseases, independently of other more specific factors [51]. Education has been traditionally described as a strong determinant of differences in health and to determine a remarkably homogeneous gradient of prevalence at the disadvantage of low educated people across age classes for many chronic diseases, in Europe [52]. In France, four social indicators have been identified, independent of age and gender, as determinants of HRQoL, including married or living with a common-law partner, the level of education, the occupational status, and the net household income [53]. Finally, access to subspecialty care (a surrogate for HCI) contributes to the known disparities in morbidity and mortality observed in some rheumatic diseases, while expediting the diagnosis and treatment of rheumatic diseases reduces disparities [54, 55].

Fourth, the RAPID-3 score was consistently associated with all the dimensions and facets scores. In contrast, corticosteroid use was negatively associated with physical function dimension and overall QoL facet scores. There is published evidence that disease activity/severity impacts HRQoL in the clinical context of RA, which has been confirmed in studies where disease activity was evaluated with the RAPID-3 [56], and where the QoL was assessed with the WHOQOL, which includes 100 items [57]. Also, in a recent survey that examined patient perspectives on prednisolone use in a cohort of RA patients, it was found that current users were older and had a longer disease duration and scored poorer patient-reported outcomes, such as higher levels of pain, more insufficient disease control, more significant disability, and poorer HRQoL, when compared to non-users [58]. Similar results had been published in SLE German patients with no glucocorticoid intake associated with better HRQoL [59].

Finally, the most consistent predictor of six-month follow-up QoL dimensions and facets scores was each corresponding baseline dimension/facet score, while (some) social determinants (years of scholarship and having a job), emotional factors (feeling bored), and biomedical aspects (RAPID 3) had an additional impact. Huang et al. [60] performed a longitudinal study intending to investigate changes in the QoL in patients receiving home-based primary care; authors applied a locally tailored health-related questionnaire and identified that scores dropped gradually from the 1^st^ year to the 5^th^ year follow-up, and baseline QoL score predicted longitudinal change in 5 years follow-up quality of life scores. In the field of rheumatic diseases, additional studies had confirmed that baseline status defined trends in terms of radiographic progression [61], disease activity [62], and disability [63].

Limitations of the study need to be addressed. We used the DASS21 to assess emotional comorbidity, which was not confirmed by a psychiatric interview; in addition, prevalence rates of depression and anxiety may be due to scale-specific case definition [64]; also, HCI was self-referred, and the outcome was not corroborated. RAPID-3 was used to assess disease activity/severity among patients with a wide variety of rheumatic diagnoses, while the scale has been validated only in RA patients. We studied a limited number of factors associated with the QoL, while others like exercise and nutritional state [59], previous COVID-19 vaccination [65], and coping skills [66] were not assessed; also, our results might be influenced by the variable selection method used. The underlying rheumatic diagnosis has been shown to be an independent predictor of HRQoL, with inflammatory myopathies patients with early disease showing the most severe impairment in both physical and mental HRQoL [67]. Control over negative emotions was not assessed, while poor control over the negative (and positive) emotions, rather than emotions themselves, had been considered a significant risk factor for a broad range of psychopathologies [68]. We conceived the patient’s QoL as an outcome, rather than a process, and the application of the WHOQOL-BREF was intended to screen and to monitor progress in individual patient care; different applications of QoL-related instruments need to be distinguished because instruments that work very well for one purpose need not necessarily be helpful when used in another context [69]. Finally, recent literature has discussed the difficulty in applying the biopsychosocial model to some clinical conditions and the need for a re-formulation [70, 71].

## Conclusions

In the current study, HCI during the COVID-19 pandemic impacted patient´s QoL dimensions, but the environment dimension, among patients with different rheumatic diseases. At patients´ reincorporation to the outpatient clinic, psycho-emotional, sociodemographic and biomedical factors were consistently associated with QoL dimensions scores. Similar factors were associated with QoL dimensions scores at six-month follow-up, in addition to the baseline QoL status, which consistently predicted QoL trajectory.

Traditional models that focus primarily on biomedical information are insufficient to assess patient’s health trajectories comprehensively. Social determinants of health and the individual’s emotional sphere should be more actively assessed and integrated into biomedical assessments to expand the scope of barriers to achieve relevant outcomes in patients with rheumatic diseases. This reinforces the idea to re-envision interdisciplinary work with integrated care teams that proactively and systematically screen patients for mental health problems or psychosocial determinants while working on a collaborative model.

## Supporting information

S1 Checklist CONSORT checklist. (PDF)

S1 Appendix COVID-19 survey. (PDF)

S1 Table Comparison of baseline characteristics among those who completed/did not complete the study. (PDF)

## References

[1] Edlund M, Tancredi LR. Quality of Life: An ideological critic. Perspect Biol Med. 1985; 28(4):591–607. doi: 10.1353/pbm.1985.0034 4034363

[2] McClimans L, Browne JP. Quality of life is a process not an outcome. Theor Med Bioeth. 2012; 33:279–292. doi: 10.1007/s11017-012-9227-z 22798191

[3] Parmenter TR. Quality of life as a concept and measurable entity. Soc Ind Res. 1994; 33:9–46.

[4] Karimi M, Brazier J. Health, Health-Related Quality of Life, and Quality of Life: What is the Difference? PharmacoEconomics. 2016; 34(7): 645–49. doi: 10.1007/s40273-016-0389-9 26892973

[5] Development of the World Health Organization WHOQOL-BREF Quality of Life Assessment the WHOQOL group. Psychol Med. 1998; 28: 5515–8. doi: 10.1017/s0033291798006667 9626712

[6] Russell A, Gulliver WP, Irvine EJ, Albani S, Dutz JP. Quality of Life in Patients with Immune-Mediated Inflammatory Diseases. J Rheumatol. 2011; 88: 7–19. doi: 10.3899/jrheum.110899 22045973

[7] Ward MM. Outcome measurement: health status and quality of life. Curr Opin Rheumatol. 2004; 16: 96–101. doi: 10.1097/00002281-200403000-00004 14770092

[8] Walker JG, Littlejohn GO. Measuring quality of life in rheumatic conditions. Clin Rheumatol. 2007; 26: 671–3. doi: 10.1007/s10067-006-0450-8 17124551 PMC1847465

[9] Beaudart C, Biver E, Bruyère O, Cooper C, Al-Daghri N, Reginster JY, et al. Assessment of Quality of Life in Musculo-Skeletal Health. Aging Clin Exp Res. 2018; 30(5): 413–18. doi: 10.1007/s40520-017-0794-8 28664458 PMC5653197

[10] Wolfe F, Michaud K, Li T, Katz RS. EQ-5D and SF-36 quality of life measures in systemic lupus erythematosus: comparisons with rheumatoid arthritis, noninflammatory rheumatic disorders, and fibromyalgia. J Rheumatol. 2010; 37: 296–304. doi: 10.3899/jrheum.090778 20032098

[11] Beresniak A, Russell AS, Haraoui B, Bessette L, Bombardier C, Duru G. Advantages and limitations of utility assessment methods in rheumatoid arthritis. J Rheumatol. 2007; 34: 2193–200. 17937471

[12] Russak SM, Croft JD Jr, Furst DE, Hohlbauch A, Liang MH, Moreland L, et al. The use of rheumatoid arthritis health related quality of life patient questionnaires in clinical practice: lessons learned. Arthritis Rheum. 2003; 49: 574–84. doi: 10.1002/art.11208 12910566

[13] Maly MR, Costigan PA, Olney SJ. Determinants of self-report outcome measures in people with knee osteoarthritis. Arch Phys Med Rehabil. 2006; 87: 96–104. doi: 10.1016/j.apmr.2005.08.110 16401446

[14] Verbrugge LM, Juarez L. Profile of arthritis disability: II. Arthritis Care Res. 2006; 55: 102–13. doi: 10.1002/art.21694 16463411

[15] Ibarra-Nava I, Cárdenas-De La Garza JA, Ruiz-Lozano RE, Salazar-Montalvo RG. Mexico and the COVID-19 response Disaster. Med Public Health Prep 2020; 14:e17–e18. 10.1017/dmp.2020.260 PMC7445449 32713412

[16] Ciurea A, Papagiannoulis E, Bürki K, von Loga I, Micheroli R, Möller B, et al. Impact of the COVID-19 pandemic on the disease course of patients with inflammatory rheumatic diseases: Results from the Swiss Clinical Quality Management cohort. Ann Rheum Dis. 2020; 80: 238–41. doi: 10.1136/annrheumdis-2020-218705 32963052

[17] Sachdev Manjit Singh B, Chuah SL, Cheong YK, Wan SA, Teh CL. Impact of lockdown on rheumatology outpatient care in the age of COVID-19. Ann Rheum Dis. 2020; August 7: annrheumdis-2020-218484. doi: 10.1136/annrheumdis-2020-218484 32769156

[18] George MD, Venkatachalam S, Banerjee S, Baker JF, Merkel PA, Gavigan K, et al. Concerns, healthcare use, and treatment interruptions in patients with common autoimmune rheumatic diseases during the COVID-19 pandemic. J Rheumatol. 2020; Nov 15: jrheum.201017. 10.3899/jrheum.201017 PMC8121899 33191284

[19] Endstrasser F, Braito M, Linser M, Spicher A, Wagner M, Brunner A. The negative impact of the COVID-19 lockdown on pain and physical function in patients with end-stage hip or knee osteoarthritis. Knee Surg Sports Traumatol Arthrosc. 2020; 28: 2435–43. doi: 10.1007/s00167-020-06104-3 32556438 PMC7299668

[20] Glintborg B, Jensen DV, Engel S, Terslev L, Pfeiffer Jensen M, Hendricks O, et al. Self-protection strategies and health behaviour in patients with inflammatory rheumatic diseases during the COVID-19 pandemic: results and predictors in more than 12 000 patients with inflammatory rheumatic diseases followed in the Danish DANBIO registry. RMD Open. 2021; 7: e001505. doi: 10.1136/rmdopen-2020-001505 33402443 PMC7786545

[21] Antony A, Connelly K, De Silva T, Eades L, Tillett W, Ayoub S, et al. Perspectives of patients with rheumatic diseases in the early phase of COVID-19. Arthritis Care Res. 2020; 72: 1189–95. doi: 10.1002/acr.24347 32526068 PMC7300883

[22] Hooijberg F, Boekel L, Vogelzang EH, Leeuw M, Boers M, van Vollenhoven R, et al. Patients with rheumatic diseases adhere to COVID-19 isolation measures more strictly than the general population. Lancet Rheumatol. 2020; 2: e583–5. doi: 10.1016/S2665-9913(20)30286-1 33106791 PMC7579459

[23] Michaud K, Wipfler K, Shaw Y, Simon TA, Cornish A, England BR, et al. Experiences of patients with rheumatic diseases in the United States during early days of the COVID-19 pandemic. ACR Open Rheumatol. 2020; 2: 335–43. doi: 10.1002/acr2.11148 32311836 PMC7264613

[24] Schmeiser T, Broll M, Dormann A, Fräbel C, Hermann W, Hudowenz O, et al. A cross sectional study on patients with inflammatory rheumatic diseases in terms of their compliance to their immunsuppressive medication during COVID-19 pandemic. Z Rheumatol. 2020; 79: 379–84. doi: 10.1007/s00393-020-00800-8 32303821 PMC7163348

[25] Khabbazi A, Kavandi H, Paribanaem R, Khabbazi R, Malek Mahdavi A. Adherence to medication in patients with rheumatic diseases during COVID-19 pandemic. Ann Rheum Dis. 2020: annrheumdis-2020-218756. 10.1136/annrheumdis-2020-218756 32895235

[26] Seyahi E, Poyraz BC, Sut N, Akdogan S, Hamuryudan V. The psychological state and changes in the routine of the patients with rheumatic diseases during the coronavirus disease (COVID-19) outbreak in turkey: a web-based cross-sectional survey. Rheumatol Int. 2020; 40: 1229–38. doi: 10.1007/s00296-020-04626-0 32572609 PMC7306572

[27] Cleaton N, Raizada S, Barkham N, Venkatachalam S, Sheeran T, Adizie T, et al. COVID-19 prevalence and the impact on quality of life from stringent social distancing in a single large UK rheumatology centre. Ann Rheum Dis. 2020; annrheumdis-2020-218236. 10.1136/annrheumdis-2020-218236 32719041

[28] Koppert TM, Jacobs JWG, Geenen R. The psychological impact of the COVID-19 pandemic on Dutch people with and without an inflammatory rheumatic disease. Rheumatology. 2020; 0: 1–7. 10.1093/rheumatology/keaa842 PMC7798513 33313870

[29] Guaracha-Basáñez GA, Contreras-Yáñez I, Hernández-Molina G, González-Marín A, Pacheco-Santiago LD, Valverde-Hernández SS, et al. Clinical and bioethical implications of health care interruption during the COVID-19 pandemic: a cross-sectional study in outpatients with rheumatic diseases. Plos One 2021. 2021;1 6 (7): e0253718. doi: 10.1371/journal.pone.0253718 34242245 PMC8270122

[30] World Medical Association. Ethical principles for medical research involving human subjects. Eur J Emerg Med. 2001; 8: 2212–3. 10.1097/00063110-200109000-00010 11587468

[31] Infante-Castañeda C, Peláez Ballestas I, Giraldo-Rodríguez L. Covid-19 y género: efectos diferenciales de la pandemia en universitarios. Revista Mexicana de Sociologí-a, 0. 2021. 10.22201/iis.01882503p.2021.0.60072.

[32] England BR, Sayles H, Mikuls TR, Johnson DS, Michaud K. Validation of the rheumatic disease comorbidity index. Arthritis Care Res. 2015; 67 (6): 8657–2. doi: 10.1002/acr.22456 25186344

[33] Pincus T, Bergman MJ, Yazici Y. RAPID3-an index of physical function, pain, and global status as "vital signs" to improve care for people with chronic rheumatic diseases. Bull NYU Hosp Jt Dis. 2009; 67 (2): 2112–5. 19583557

[34] Daza P, Novy D, Stanley M, Averill P. The Depression Anxiety Stress Scale-21: Spanish Translation and Validation with a Hispanic Sample. J Psychopathol Behav Assess. 2002; 24: 1952–05.

[35] Weiss DS, Marmar CR. The impact of event scale–revised. In: Wilson JP, Keane TM, editors. Assessing psychological trauma and PTSD. New York: Guilford Press; 1997. pp. 399–411.

[36] Báguena MJ, Villarroya E, Beleña Á, Díaz A, Roldán C, Reig R. Propiedades psicométricas de la versión española de la escala revisada de Impacto del Estresor (EIE-R). Análisis Modif. Conduct. 2001; 27, 581–604.

[37] Lovibond SH, Lovibond PF. Manual for the Depression Anxiety & Stress Scales. 2^nd^ ed. Sydney: Psychology Foundation; 1995.

[38] de Mol M, Visser S, Aerts JGJV, Lodder P, de Vries J, den Oudsten BL. Satisfactory results of a psychometric analysis and calculation of minimal clinically important differences of the World Health Organization quality of life-BREF questionnaire in an observational cohort study with lung cancer and mesothelioma patients. BMC Cancer. 2018; 18: 1173. doi: 10.1186/s12885-018-4793-8 30477456 PMC6260568

[39] Engel GL. The need for a new medical model: a challenge for biomedicine. Science. 1977; 196: 129–36. doi: 10.1126/science.847460 847460

[40] Michaud K, Vera-LLonch M, Oster G. Mortality Risk by Functional Status and Health-related Quality of Life in Patients with Rheumatoid Arthritis. Rheumatology. 2012; 39:1; doi: 10.3899/jrheum.110491 22089466

[41] Guyatt GH, Bombardier C, Tugwell PX. Measuring disease-specific quality of life in clinical trials. CMAJ. 1986; 134:889–95. 3955482 PMC1490966

[42] Matcham F, Scott IC, Rayner L, Hotopf M, Kingsley GH, Norton S, et al. The impact of rheumatoid arthritis on quality-of-life assessed using the SF-36: A systematic review and meta-analysis. Semin Arthritis Rheum. 2014; 44: 123–30. doi: 10.1016/j.semarthrit.2014.05.001 24973898

[43] Picavet HSJ, Hoeymans N. Health related quality of life in multiple musculoskeletal diseases: SF-36 and EQ-5D in the DMC3 study. Ann Rheum Dis. 2004; 63:723–9. doi: 10.1136/ard.2003.010769 15140781 PMC1755044

[44] Skevington S.M., Lotfy M.2 & O’Connell K.A. The World Health Organization’s WHOQOL-BREF quality of life assessment: Psychometric properties and results of the international field trial. A Report from the WHOQOL Group. Qual Life Res. 2004; 13: 299–310. doi: 10.1023/B:QURE.0000018486.91360.00 15085902

[45] Lancet The. COVID-19 in Latin America: a humanitarian crisis. Lancet 2020; 396: 1463. doi: 10.1016/S0140-6736(20)32328-X 33160550

[46] Pope JE. What does the COVID-19 pandemic mean for rheumatology patients? Curr Treat Options Rheumatol. 2020; 30: 1–4. doi: 10.1007/s40674-020-00145-y 32355607 PMC7191545

[47] Brown BA, Goodman FR, Disabato DJ, Kashdan TB, Armeli S, Tennen H. Does negative emotion differentiation influence how people choose to regulate their distress after stressful events? A four-year daily diary study. Emotion. 2021; doi: 10.1037/emo0000969 33829837

[48] Cutuli D. Cognitive reappraisal and expressive suppression strategies role in the emotion regulation: an overview on their modulatory effects and neural correlates. Front Syst Neurosci. 2014;8:175. doi: 10.3389/fnsys.2014.00175 25285072 PMC4168764

[49] Mennin DS, Holaway RM, Fresco DM, Moore MT, Heimberg RG. Delineating components of emotion and its dysregulation in anxiety and mood psychopathology. Behav Ther. 2007; 38 (3): 284–302. doi: 10.1016/j.beth.2006.09.001 17697853

[50] Gross JJ, John OP. Individual differences in two emotion regulation processes: implications for affect, relationships, and well-being. J Pers Soc Psychol. 2003; 85 (2): 348–62. doi: 10.1037/0022-3514.85.2.348 12916575

[51] Guillemin AF, Carruthers E, Li LC. Determinants of MSK health and disability- Social determinants of inequities in MSK health. Best Pract Res Clin Rheumatol. 2014; 28 (3): 411–33. doi: 10.1016/j.berh.2014.08.001 25481424

[52] Dalstra JA, Kunst AE, Borrell C, Breeze E, Cambois E, Costa G, et al. Socioeconomic differences in the prevalence of common chronic diseases: an overview of eight European countries. Int J Epidemiol. 2005; 34 (2): 316e26. doi: 10.1093/ije/dyh386 15737978

[53] Kivits J, Erpelding ML, Guillemin F. Social determinants of health-related quality of life. Rev Epidemiol Sante Publique. 2017; 65 (2): 137–48. doi: 10.1016/j.respe.2017.01.001 23849946

[54] Lennep DS, Crout T, Majithia V. Rural health issues in rheumatology: A review. Curr Opin Rheumatol. 2020; 32: 119–25. doi: 10.1097/BOR.0000000000000694 31913162

[55] Williams EM, Ortiz K, Browne T. Social Determinants of Health, the Chronic Care Model, and Systemic Lupus Erythematosus. Int J Chronic Dis. 2014; 2014:361792. doi: 10.1155/2014/361792 26464854 PMC4590929

[56] Qorolli M, Rexhepi B, Rexhepi S, Mustapić M, Doko I, Grazio S. Association between disease activity measured by RAPID3 and health related quality of life in patients with rheumatoid arthritis. Rheumatol Int. 2019; 39 (5): 827–34. doi: 10.1007/s00296-019-04258-z 30847560

[57] Chiu YM, Lai MS, Lin HY, Lang HC, Lee LJ, Wang JD. Disease activity affects all domains of quality of life in patients in patients with rheumatoid arthritis and is modified by disease duration. Clin Exp Rheumatol. 2014; 32: 898–903. 25189095

[58] Venter G, Tieu J, Black R, Lester S, Leonardo N, Whittle SL, et al. Perspectives of Glucocorticoid Use in Patients with Rheumatoid Arthritis. ACR Open Rheumatol. 2021; 3 (4): 231–38. doi: 10.1002/acr2.11234 33609083 PMC8063143

[59] Vordenbäumen S, Brinks R, Sander O, Chehab G, Lozitiello-Kiroudis G, Acar H, Richter J, et al. Determinanten gesundheitsbezogener Lebensqualität bei systemischem Lupus erythematodes: eine monozentrische, retrospektive Langzeitobservationsstudie in Deutschland [Determinants of health-related quality of life in systemic lupus erythematosus: a monocentric, retrospective long-term observational study in Germany]. Z Rheumatol. 2019; 78 (9): 813–19. doi: 10.1007/s00393-019-00691-4 31468165

[60] Huang CH, Umegaki H, Kamitani H, Asai A, Kanda S, Maeda K, et al. Change in quality of life and potentially associated factors in patients receiving home-based primary care: a prospective cohort study. Huang et al. BMC Geriatrics. 2019; 19: 21. doi: 10.1186/s12877-019-1040-3 30678632 PMC6345012

[61] Krause D, Gabriel B, Herborn G, Braun J, Rau R. Radiologic damage at baseline predicts patient-related outcomes 18 years after the initiation of methotrexate therapy in patients with severe rheumatoid arthritis. Clin Exp Rheumatol. 2015; 33 (5): 611–16. 26315962

[62] Pongratz G, Frieser R, Brinks R, Schneider M, Hartung W, Fleck M, et al. Association between autoantibody level and disease activity in rheumatoid arthritis is dependent on baseline inflammation. Clin Exp Rheumatol. 2020; 38(4): 691–98. 31858962

[63] Allanore Y, Bozzi S, Terlinden A, Huscher D, Amand C, Soubrane C, et al. Health Assessment Questionnaire-Disability Index (HAQ-DI) use in modelling disease progression in diffuse cutaneous systemic sclerosis: an analysis from the EUSTAR database. Arthritis Res Ther. 2020; 22 (1): 257. doi: 10.1186/s13075-020-02329-2 33115544 PMC7592571

[64] Covic T, Cumming SR, Pallant JF, Manolios N, Emery P, Conaghan PG, et al. Depression and Anxiety in Patients with Rheumatoid Arthritis: Prevalence rates based on a comparison of the Depression, Anxiety and Stress Scale (DASS) and the Hospital, Anxiety and Depression Scale (HADS). BMC Psychiatry. 2012; 24(12):6. 10.1186/1471-244X-12-6 PMC3285517 22269280

[65] Boekel L, Kummer LY, van Dam KPJ, Hooijberg F, van Kempen Z, Vogelzang EH, et al. Adverse events after first COVID-19 vaccination in patients with autoimmune diseases. Lancet Rheumatol. 2021; doi: 10.1016/S2665-9913(21)00181-8 34179831 PMC8213359

[66] Wang XA, Duculan R, Mancuso CA. Coping Mechanisms Mitigate Psychological Stress in Patients With Rheumatologic Diseases During the COVID-19 Pandemic. J Clin Rheumatol. 2021; doi: 10.1097/RHU.0000000000001757 34054073

[67] Greenfield J, Hudson M, Vinet E, Fortin PR, Bykerk V, Pineau CA, et al. Canadian Scleroderma Research Group and Canadian Inflammatory Myopathy Study Group. A comparison of health-related quality of life (HRQoL) across four systemic autoimmune rheumatic diseases (SARDs). PLoS One. 2017; 12 (12): e0189840. doi: 10.1371/journal.pone.0189840 29261752 PMC5736192

[68] Johnson SL, Elliott MV, Carver CS. Impulsive Responses to Positive and Negative Emotions: Parallel Neurocognitive Correlates and their Implications. Biol Psychiatry. 2020; 87(4): 338–49. doi: 10.1016/j.biopsych.2019.08.018 31668478 PMC7012660

[69] Fitzpatrick R. The measurement of health status and quality of life in rheumatological disorders. Bailliere’ s Clinical Rheumatology—Vol. 7, No. 2, June 1993, ISBN 0-7020-1710-8. doi: 10.1016/s0950-3579(05)80091-3 8334714

[70] Pincus T, Kent P, Bronfort G, Loisel P, Pransky G, Hartvigsen J. Twenty-Five Years with the Biopsychosocial Model of Low Back Pain—Is it Time to Celebrate? A Report from the Twelfth International Forum for Primary Care Research on Low Back Pain. Spine. 2013; 38 (24): 2118–23. doi: 10.1097/BRS.0b013e3182a8c5d6 23970112

[71] Searight HR. The Biopsychosocial Model: ‘‘Reports of My Death Have Been Greatly Exaggerated”. Cult Med Psychiatry. 2016; 40: 289–98. doi: 10.1007/s11013-015-9471-6 26374750

