# Supplementary material for: Quality of life of patients with rheumatic diseases during the COVID-19 pandemic: The biopsychosocial path
Source: PLoS One. 2022 Jan 18;17(1):e0262756. doi: 10.1371/journal.pone.0262756 (PMC8765619; doi:10.1371/journal.pone.0262756)
Supplement: S1 Appendix — (PDF) [file pone.0262756.s002.pdf]

COVID-19 SURVEY (ENGLISH VERSION)

The Immunology and Rheumatology Department of the Instituto Nacional de Ciencias Médicas y de la Nutrición Salvador Zubirán, is interested in knowing some aspects about your general health and your rheumatic disease course during the COVID-19 pandemic. We also want to know how you have faced those health problems during the period when the Institution was a dedicated COVID-19 hospital.

Please complete the following information:

Date:___________________________

Answer the following questions, marking with an X the response option that best suits your case (it might be more than one answer).

For all the questions, the considered period of time is from March 17, 2020 (the Institution was declared a dedicated COVID-19 hospital and the outpatient clinic was closed) to the outpatient clinic (partially) reinstitution. We have called this period of time "COVID-19 pandemic".

1. **During the COVID-19 pandemic, has Institutional health care provision for your rheumatic disease been affected in any way?**

   ☐ No

   ☐ Yes

   If you answered YES, then select reasons with a cross (there could be more than one possible reason)

**1.a** I decided not to attend my schedule appointment during the pandemic	
**1.b** The Institution canceled my appointment	
**1.c** The Institution rescheduled my appointment to a later date	
**1.d** I required an extra appointment and could not schedule it	
**1.e** I required an emergency consultation but it was not available	
**1.f** Other reason, please specify	

2. **During the COVID-19 pandemic, have you required health care assistance?**

☐ No

☐ Yes, because of my rheumatic disease

Specify the reason: ________________________________

☐ Yes, for a reason other than my rheumatic disease

Specify the reason: ________________________________

3. **During the COVID-19 pandemic, have you required hospitalization?**

☐ No

☐ Yes, because of my rheumatic disease

Specify the reason: ________________________________

☐ Yes, for a reason other than my rheumatic disease

Specify the reason: ________________________________

4. **If you have required health care assistance and/or hospitalization for REASONS RELATED TO YOUR RHEUMATIC DISEASE, whom have you consulted or where did you receive it?**

**4.a Health care assistance**	
**4.a1 Who did you consulted?**	
	General practitioner
	Rheumatologist
	Internist
Other, specify	
**4.a2 Where did you received the health care assistance?**	
	Instituto Nacional de Ciencias Médicas y Nutrición
	Private hospital
	Other public hospital
Other hospital, please specify:	
Not applicable/ I did not require health care assistance	☐

4.b HOSPITALIZATION	
**4.b1 Where were you hospitalized?**	
	Private hospital
	Other public hospital
Other hospital, please specify:	
Not applicable/ Did not need hospitalization ☐	

5. **During the COVID-19 pandemic, have you required to contact a physician from the Immunology and Rheumatology Department? (you may choose more than one option)**

☐ I have not required it

☐ Yes, due to problems with my rheumatic disease

☐ Yes, due to doubts regarding my rheumatic disease-related treatment

☐ Yes, due to concerns about the COVID-19 pandemic

☐ Yes, due to other reasons

Write them down: ______________________________________

6. **Regarding the previous question, were you able to contact someone from the Immunology and Rheumatology Department?**

☐ Yes, I could

☐ No, I couldn´t

☐ I didn't have the need

7. **In case you had communicated with the Immunology and Rheumatology Department, what was the way used? (you can choose more than one option)**

☐ Not applicable

☐ Email assigned to the Immunology and Rheumatology Department

☐ Social Networks

☐ Telephone

☐ Other ways

Write them: ______________________________________

8. During the COVID-19 pandemic, the personnel from the Immunology and Rheumatology Department and/or from the Institution might have communicated with you. We are interested to knowing who made the contact and what was the purpose?

☐ Yes

☐ No

☐ Not applicable

☐ I believe that the Institution staff cannot localize me

8.b Were you able to identify the purpose?

☐ Yes

☐ No

☐ Not applicable

8.c Select one of the following options, only if you have had communication with someone from the Immunology and Rheumatology Department. (YOU CAN CHOOSE MORE THAN ONE OPTION)

	**8.c.1** The Institution contacted me to reschedule my medical and/or laboratory appointment
	**8.c.2** The Institution contacted me to give me a medical consultation and/or laboratory results, not from the rheumatology specialty, (phone-consultation)
	**8.c.3** The Immunology and Rheumatology Department contacted me to reschedule my rheumatology appointment and/or the corresponding laboratory tests
	**8.c.4** The Immunology and Rheumatology Department contacted me to give me my rheumatology appointment and/or laboratory results (phone-consultation)
	**8.c.5** I contacted the Institution for questions about my medical appointments and/or laboratory tests
	**8.c.6** I contacted the Immunology and Rheumatology Department to answer concerns related to my rheumatology appointment and and/or laboratory tests
	**8.c.7** I contacted the Institution to answer questions about the COVID-19 pandemic
	**8.c.8** I contacted the Immunology and Rheumatology Department to answer questions about the COVID-19 pandemic

	**8.c.9** I contacted the Institution to answer doubts about my rheumatic disease and/or rheumatic disease-related treatment
	**8.c.10** I contacted the Immunology and Rheumatology Department to answer doubts about my rheumatic disease and/or rheumatic disease-related treatment
	**8.c.11** I could not communicate with the Institution
	**8.c.12** I could not communicate with the Immunology and Rheumatology Department
	**8.c.13** Other reasons, please specify :
	I did not have the need to communicate with the Institution or with the Immunology and Rheumatology Department

9. **In case you have communicated with personnel from the Institution (not in particular with staff from the Immunology and Rheumatology Department), what was the way used? (YOU CAN CHOOSE MORE THAN ONE OPTION)**

- ☐ Not applicable
- ☐ Email assigned to the Immunology and Rheumatology Department
- ☐ Social Networks
- ☐ Telephone
- ☐ Other ways

  Write them: ______________________________________

10. **If you have communicated with staff from the Immunology and Rheumatology Department, how helpful was it?**

**Not helpful**	**Not very helpful**	**Moderately helpful**	**Quite helpful**

☐ I did not communicate

11. **During the COVID-19 pandemic, have you made any changes to your rheumatic disease-related treatment?**

☐ I do not have any treatment indicated
☐ I have not made any changes to my medications
☐ Yes, I have made changes, indicated by a rheumatologist

☐ Yes, I have made changes, indicated by another doctor

☐ Yes, I have made changes, by my own decision

☐ Yes, I have made changes, because of lack of money to buy them

☐ Yes, I have made changes, because shortage in medication supply

Specify: Which medication(s) were you unable to obtain?

____

☐ Yes, I have made changes, for other reasons

Write reason(s): ____

12. **If you have changed the rheumatic disease-related treatment, could you mention what the change was and the drug affected?**
FOR EACH OPTION SELECTED, WRITE DOWN THE NAME OF THE MEDICATION

☐ I have not made any changes

☐ I totally failed

☐ I suspended for a few days

☐ I increased the doses

☐ I decreased the doses

☐ I took other medications that I didn´t have indicated

☐ Other changes (write them down)

____

____

13. **If you had a problem getting your medication, what was the reason?**

FOR EACH OPTION SELECTED, WRITE DOWN THE NAME OF THE MEDICATION ON THE RIGHT

- ☐ Not applicable
- ☐ The drug store did not have the medication
- ☐ It was not delivered by my social security
- ☐ I didn't have money to buy it
- ☐ Other

  Write them: ____________________________________

14. **Have you and/or any family member with who you live or visit frequently, been diagnosed with COVID-19?**

- ☐ No, no one
- ☐ Yes, my self was diagnosed
- ☐ Yes, some of my family members were diagnosed
- ☐ Yes, several of us were diagnosed, including my self

15. **Have you or any family member with who you live or visit frequently, been tested for COVID-19?**

- ☐ No one was tested because there was no reason to do it
- ☐ No, diagnosis of COVID-19 disease was performed only with the symptoms
- ☐ Yes, the test for COVID-19 disease was performed (sampling by the nose or throat)
- ☐ Yes, the imaging study was performed to view the lungs (X-ray or CT scan)

16. **What were the symptoms you experienced (you may choose more than one option)**

☐ I had no symptoms or discomfort from COVID-19 disease

☐ Fever greater than 38°	☐ Muscle pain	☐ Difficulty perceiving odors
☐ Dry cough	☐ Throat pain	☐ Difficult to breath
☐ Diarrhea	☐ Vomiting	☐ Extreme fatigue
☐ Chest pain	☐ Headache	☐ Nasal congestion

☐ Other, write them: ____

17. **During the COVID 19 pandemic, how much did you follow recommendations to stay home as long as possible?**

**Always**	**Almost always**	**Sometimes**	**Almost never**	**Never**

18. **In case you have answered the previous question (number 17), with one of the following "Sometimes", "Almost never" or "Never", what was the reason?**

☐ Because of my work

☐ Because of having family obligations

☐ I did not consider it was necessary

☐ I didn’t know I had to stay home

☐ Other reasons (please write them down below)

____

☐ Not apply because I stayed home “Always” or “Almost Always”

19. **During the COVID-19 pandemic, what were your biggest concerns related to your overall health or rheumatic disease?**

Related to your **GENERAL HEALTH**	Related to your **RHEUMATIC DISEASE**

☐ I didn't worry about my overall health ☐ I didn't worry about my rheumatic disease

20. **During the COVID-19 pandemic, how much help did you get from the Immunology and Rheumatology Department to resolve and/or address these health concerns?**

**Not helpful**	**Somewhat helpful**	**More or less helpful**	**Quite helpful**	**Very helpful**

21. **During the COVID-19 pandemic, the Institution was a dedicated COVID-19 hospital and temporarily canceled routine care for all outpatients. How did you feel about it?**

____

____

22. **How would you rate the seriousness of the COVID-19 pandemic in Mexico?**

☐ Very high

☐ High

☐ Moderate

☐ Low

☐ No seriousness

23 How likely do you think you will get the SARS-CoV-2 infection?

☐ Very high

☐ High

☐ Low

☐ Very low

☐ I have already caught it

24 Regarding COVID-19 pandemic, how much has it made you feel …

	Very much intensity	Much intensity	Middle intensity	Low intensity	Nothing
**24.1 Anxious**					
**24.2 Worried**					
**24.3 Fearful**					
**24.4 Alertness**					
**24.5 Depressed**					
**24.6 Confused**					
**24.7 Alarmed**					
**24.8 Isolated**					
**24.9 Discriminated against**					
**24.10 Bored**					

25 Did the COVID-19 pandemic affect the family-members relationship?

☐ No

☐ Yes, positively

☐ Yes, negatively

26 Did the COVID-19 pandemic had a negative family economic impact?

- ☐ No
- ☐ Yes

27 If you answered yes to the previous question, what were the reasons?

- ☐ Unemployment
- ☐ Closing of family or non-family businesses
- ☐ Loss of work
- ☐ Lack of payment (for example, due to the closure of institutions)
- ☐ Other. Specify: ____

28 How do you rate the risk of getting SARS-CoV-2 infection at outpatient clinic reopening?

- ☐ Very high
- ☐ High
- ☐ Moderate
- ☐ Low
- ☐ I do not know

THANK YOU FOR YOUR PARTICIPATION
