# Supplementary material for: Quality of life of patients with rheumatic diseases during the COVID-19 pandemic: The biopsychosocial path
Source: PLoS One. 2022 Jan 18;17(1):e0262756. doi: 10.1371/journal.pone.0262756 (PMC8765619; doi:10.1371/journal.pone.0262756)
Supplement: S1 Table — (PDF) [file pone.0262756.s003.pdf]

Supplementary table. Comparison of baseline characteristics among those who completed/did not complete the study.

	Patients who did not completed study follow-up N=29 (10.5%)	Patients who completed study N= 247 (89.5%)	p
**Socio-demographic characteristics**			
Age, years	45(32.5-57)	44(33-55)	0.878
Females*	26(89.7)	203(82.2)	0.311
Years of scholarship	12(9-16.5)	12(9-17)	0.733
Living together*	10(34.5)	121(49)	0.139
Formal and non-formal job*	12(41.4)	113(45.7)	0.655
Access to Social Security benefits*	9(31)	87(35.2)	0.654
Middle-low socioeconomic level*	29(100)	220(89.1)	0.061
**Rheumatic disease characteristics**			
Non-RA diagnosis*	10(34.5)	84(34)	1
Disease duration, years	14(6.5-19)	11(6-19)	0.564
Corticosteroid use*	12(41.4)	126(51)	0.326
Immunosuppressive drug use*	21(72.4)	181(73.3)	0.921
Antimalarial use*	14(48.3)	88(35.6)	0.182
Rheumatic disease comorbidity index score	0(0-1)	0(0-1)	0.659
Rheumatic disease comorbidity index score ≥1*	9(31)	99(40.1)	0.345
Substantial disease activity level*[1]	4(13.8)	44(17.8)	0.589
Clinical deterioration*	7(24.1)	57(23.1)	0.898
Adequate control of the rheumatic disease*	19(65.5)	159(64.4)	0.903
RAPID-3 score	5.5(1.4-14.75)	6.2(1.5-11.4)	0.754
**QoL**			

Physical health dimension score (0-100)	50(38-63)	56(44-63)	0.527
Psychological health dimension score (0-100)	63(47-69)	63(50-75)	0.685
Social relationships dimension score (0-100)	56(47-75)	56(44-75)	0.969
Environment dimension score (o-100)	56(47-63)	56(50-69)	0.473
Overall quality of life facet score (1-5)	3(3-4)	3(3-4)	0.881
General health facet score (1-5)	3(2.4)	3(2-4)	0.817
**Psychological comorbidity**			
Depression subscale score	1(0-3.5)	1(0-4)	0.625
Depression*	6(20.7)	28(11.3)	0.147
Anxiety subscale score	1(0-3.5)	1(0-4)	0.546
Anxiety*	7(24.1)	44(17.8)	0.406
Stress subscale score	4(1-8)	3(1-7)	0.842
Stress*	8(27.6)	31(12.6)	0.028
IES-R score	11(1.5-26.5)	7(3-21)	0.787
Post-traumatic distress*	3(10.3)	33(13.6)	0.778
**Negative emotions (Very much intensity/much intensity) ***			
Anxious (24MD)	10(43.5)	83(36.2)	0.493
Worried (12MD)	18(66.7)	115(48.5)	0.074
Fearful (26 MD)	12(48)	71(31.6)	0.098
Alertness (20 MD)	18(66.7)	147(64.2)	0.799
Depressed (21 MD)	5(20)	45(19.6)	0.959
Confused (28 MD)	6(24)	33(14.8)	0.231
Alarmed (27 MD)	11(45.8)	73(32.4)	0.187
Isolated (28 MD)	14(58.3)	107(47.8)	0.325
Discriminated against (32 MD)	2(9.1)	13(5.9)	0.547
Bored (29 MD)	11(45.8)	53(23.8)	0.019
**Patient´ perception of risk for SARS-CoV-2 infection component survey***			
(Very high/High) Patient' perception of the pandemic seriousness in Mexico	28(96.6)	236(96.3)	0.951

(Very high/High) Patient' risk perception of SARS-CoV-2 infection	15(51.7)	118(48.2)	0.717
(Always, most of the time) Patient´ follow-up of physical distance recommendation	28(96.6)	221(89.5)	0.225
(Very high/High) Patient' risk perception of SARS-CoV-2 infection at their reincorporation to the OCDIR	17(58.6)	99(40.2)	0.058
Negative family economic impact attributed to COVID-19 pandemic	27(93.1)	187(76.3)	0.039
COVID-19 impact on the family-members relationship[1]	2(1-3)	2(2-3)	0.407

**Number (%) of patients, data presented as median (IQR) unless otherwise indicated. RA=Rheumatoid Arthritis. HCI=Health Care Interruption. RAPID-3 score= Routine Assessment of Patients Index Score-3.[1]Patients with at least moderate disease activity level according to physician evaluation.* HCI=Health Care Interruption. MD=Missing data). [1]From 1-3, where 1=negative impact, 2=neither positive nor negative and 3=positive impact.
